# Pyro-phototronic effect enhanced broadband photodetection based on CdS nanorod arrays by magnetron sputtering†

**DOI:** 10.1039/d2ra07314e

**Published:** 2022-12-09

**Authors:** Lu Li, Dingshan Zheng, Yan Xiong, Cheng Yu, Hong Yin, Xiangxiang Yu

**Affiliations:** School of Physic and Optoelectronic Engineering, Yangtze University Jingzhou 434023 P. R. China yuxx518@126.com; School of Geography Science and Geomatics Engineering, Su Zhou University of Science and Technology No. 99 Xuefu Road SuZhou 215009 P. R. China; School of Chemistry and Chemical Engineering, Hunan Institute of Science and Technology Yueyang 414006 PR China yin@hust.edu.cn; International Iberian Nanotechnology Laboratory (INL) Avenida Mestre Jose Veiga 4715-330 Braga Portugal

## Abstract

In this work, self-powered photodetectors (PDs) based on RF magnetron sputtering-fabricated CdS nanorod arrays and polished Si substrates were prepared for the first time. By introducing the pyro-phototronic effect of wurtzite CdS, the self-powered PDs exhibit a broadband response range from UV (365 nm) to IR (1310 nm) at zero bias, even beyond the bandgap limit of the material. Both the photoresponsivity and specific detectivity are also enhanced by 23.3 times compared with those only based on the photovoltaic effect. In addition, the rise and fall times of self-powered PDs are 70 μs and 90 μs under 980 nm laser illumination. This research not only expands the application of CdS nanostructures in the field of pyro-phototronics, but also greatly enriches the preparation methods of CdS based pyro-phototronics materials.

## Abstract 2

In this work, self-powered photodetectors (PDs) based on RF magnetron sputtering-fabricated CdS nanorod arrays and polished Si substrates were prepared for the first time.

## Graphical abstract

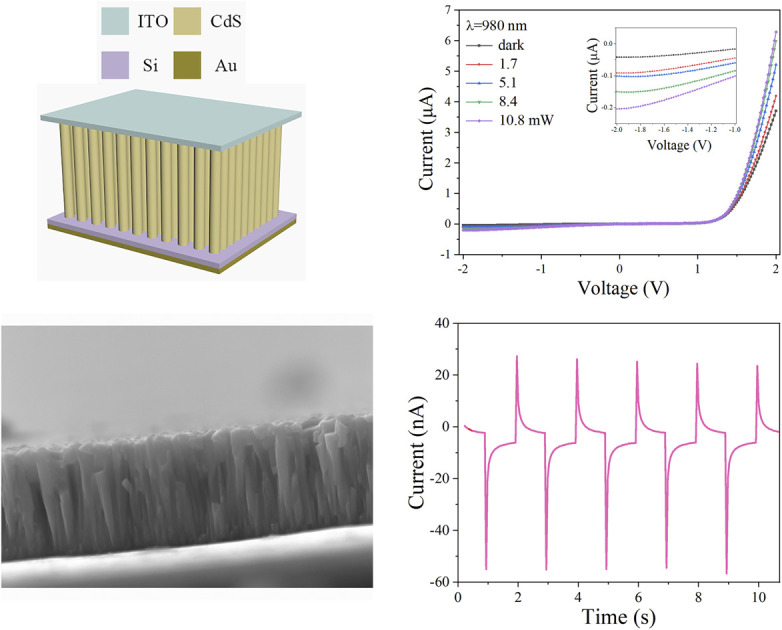

## 1. Introduction

In recent years, with the development of automotive imaging systems, high-precision electronic eyes, photocatalytic reactions and other technologies, the demand for high-performance photodetectors has become increasingly obvious. Ultra-broadband photodetectors with spectral responses from ultraviolet to infrared due to their excellent characteristics have received extensive attention.^1–4^ At the same time, self-powered heterojunction photodetectors, which can operate without external energy have become the mainstay of current research for the betterment of the next generation of nanodevices. Under the existing conditions, the current requirements for this kind of nanodevice are that the size of the devices and the energy consumption should be smaller than before, and the application range should be expanded.^5,6^ As we all know, mainstream photodetectors are concentrated in the ultraviolet and visible light bands, and there is still a bottleneck in the preparation of high-performance infrared photodetectors.^7,8^ Although narrow bandgap semiconductor materials have excellent performance in infrared absorption, conventional narrow bandgap semiconductor materials, such as InP and InAs, are still limited in the application in infrared photodetection. For photovoltaic PDs, low-temperature cooling is usually required to obtain better performance due to the influence of thermal noise. Similarly, for photoconductive PDs, an additional bias voltage device is required to start operation.^9–13^ Therefore, research on broadband self-powered photodetectors is important.

As an important third-generation semiconductor, CdS is a pyroelectric material with a non-centrosymmetric wurtzite crystal structure. When the temperature of the material changes, the properties of the material also change accordingly. When the material is irradiated with lasers of different wavelengths, the temperature of the CdS nanorods will change due to the photothermal effect, and the temperature rise of the CdS nanorods can cause the polarization of pyroelectric charges at both ends.^14–17^ Through the coupling of the pyroelectric effect, photoelectric effect and semiconductor properties, the pyro-phototronic effect can modulate the built-in field of the PN junction and improve the device performance.^18–23^ Therefore, it is imminent to investigate the applications of pyro-phototronics effect in the field of infrared photodetection.^24–27^

By intensively investigating this non-centrosymmetric property of CdS, Dai *et al.* reported self-powered PDs based on the combination of CdS nanorods and silicon.^28^ They observed that the temperature change induced by laser irradiation on CdS produces a polarization electric field in the *c*-axis of CdS nanorods. Earlier in 2020, Zhang *et al.* modified CdS nanorods with the organic molecule 2-mercaptobenzimidazole to greatly improve the pyroelectric properties of CdS and enhance the separation of pyroelectrically induced charges.^29^ Later in the same year, Yu *et al.* fabricated the SnS nanosheets coated CdS nanorods, and the pyroelectric properties arising from CdS nanorods can effectively tune the behavior of photogenerated carriers at low temperature.^30^ These results show that the ratio of pyroelectric current to photocurrent can be increased to 400% under 650 nm illumination at a temperature of 130 K. However, in previous reports, CdS nanorod arrays were basically synthesized by hydrothermal method, which has several limits. Firstly, the hydrothermal synthesis of CdS nanorods needs to consider the lattice matching of the substrate. The lattice constant of the substrate must match the nanorods, which largely limits the types of substrates. Secondly, the CdS nanorods synthesized by hydrothermal method are grown in a closed and high-pressure environment, which is unsafety. Finally, the length of CdS nanorods is difficult to adjust and the general length is always about 700 nm. These limitations restrict the further development of CdS nanorods in the photoelectrical fields. In this paper, we have introduced magnetron sputtering to prepare CdS nanorods with and without pyro-phototronics effect and the results show that magnetron sputtering has better application flexibility than the hydrothermal method.^1,31^

During the experiment, we fabricated a self-powered photodetector based on CdS nanorod arrays and p-Si by the RF magnetron sputtering method. PN junction is formed between the CdS nanorod array and Si, and the device displays a significant rectification phenomenon. In addition, the performance of the prepared photodetector was tested from UV (365 nm) to IR (1310 nm) band at zero bias voltage and the response time was also unexpectedly fast. When the device was irradiated with a 980 nm laser, the rise and fall times of the self-powered photodetector, obtained by plugging in an oscilloscope and a current amplifier, were 70 μs and 90 μs. At the same time, the peak-to-peak current, specific responsivity *R*, and detection rate D* were all improved by 23.3 times under the action of pyro-phototronic effect. Under different wavelengths of laser irradiation, the performance of the device under the action of pyroelectric polarization charges has been improved to different degrees. For comparation, due to the flexibility of magnetron sputtering, we also prepared the traditional self-powered detector based on Si/CdS heterojunction without pyro-phototronics effect by changing experimental parameters. This systematic study has not only extended the application of CdS in photoelectric areas but also indicated the direction for the realization of pyro-phototronics effect based on semiconductor materials with wurtzite structure.

## 2. Experimental section

### 2.1. Fabrication process of p-Si/n-CdS heterojunction photodetector

Firstly, the p-type single-sided polished silicon wafer (100, B-doped, 1–10 Ω cm, thickness of 500 μm) was ultrasonically cleaned in acetone, deionized water, and absolute ethanol for 15 min, respectively. Next, the silicon wafer is put into a hydrofluoric acid solution to remove the native oxide layer on the surface, and then rinsed with deionized water several times and blown dry to obtain a clean silicon wafer. Finally, the CdS nanorod array was deposited on the p-type silicon substrate by radio frequency magnetron sputtering at a sputtering power of 100 W, a chamber pressure of 0.5 Pa, and a substrate temperature of 300 °C for 60 minutes. Subsequently, a thin ITO layer is deposited on the CdS film using RF magnetron sputtering as the transparent top electrode and a thin layer of gold is evaporated by a small ion sputterer as the bottom electrode. The size of the electrode is determined by a self-made metal mask, and the effective area of the PN junction of the device is about 0.8 mm^2^.

### 2.2. Characterization and measurement

The crystal structure and microscopic structures were characterized and examined by XRD (PANalytical Empyrean, Cu Kα Radiation) and FESEM (TESCAN MIRA3), respectively. The performances of the device were tested on the platform of Vacuum Probe and Keithley 4200A-SCS semiconductor parameter analyzer. The light source is provided by a laser photodiode of different wavelengths.

## 3. Results and discussion

### 3.1. Design and characterization of p-Si/n-CdS nanorod array PDs

**Fig. 1:**
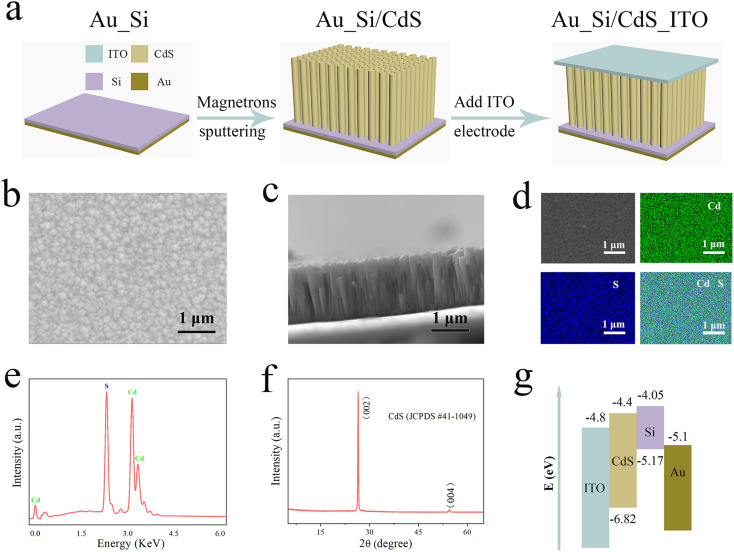
Structure and characterization of the self-powered p-Si/n-CdS heterostructure PDs. (a) Schematic demonstration of the preparing process of the self-powered p-Si/n-CdS heterostructure PDs. (b and c) Top view and cross-sectional SEM image of the as-fabricated CdS nanorod arrays. (d and e) SEM image and EDS mappings (d), EDS spectrum (e). (f) XRD pattern of CdS nanorod arrays. (g) Energy band diagram of the self-powered PDs.

The fabrication process of self-powered p-Si/n-CdS heterostructure PDs is shown in Fig. 1a. As mentioned in previous reports, although photodetectors based on pyroelectric materials such as topological insulators show broadband detection and excellent performance up to terahertz, the preparation process of such materials requires expensive equipments.^32,33^ Therefore, it is more meaningful to study self-powered broadband photodetectors based on traditional semiconductor materials with thermoelectric properties. We designed a sandwich-like structure and introduced a simple method to manufacture self-powered p-Si/n-CdS heterostructure PDs, and the corresponding patterns are shown in Fig. 1a. Firstly, a layer of gold is deposited on the back side of 500 μm thick single-sided polished silicon wafer by thermal evaporation as the bottom electrode. Then, the CdS nanorod array with a height of about 1 μm is deposited on the silicon wafer by RF magnetron sputtering to form a heterojunction. Finally, a layer of transparent electrode ITO was deposited on the top of the CdS nanorod array with a metal mask by RF magnetron sputtering as the top electrode. Fig. 1b and c show the top and cross-sectional field-emission scan electron microscopy (SEM) images of the CdS nanorod array. It can be seen that a dense and well-arranged array of CdS nanorod grows on the silicon wafer, with an average length of about 1 μm. Fig. 1d shows the SEM and EDS mappings on the top surface of the CdS nanorod array, where SEM indicates that the product has a flat surface and EDS mappings indicate that the Cd and S elements are evenly distributed. EDS spectrum in Fig. 1e further confirms that the elemental composition of the as-prepared product and the stoichiometric ratio of CdS is close to Cd : S = 1 : 1. For more accurate data, the crystal structure of the CdS nanorod arrays was determined by X-ray diffraction (XRD), and the measured result is shown in Fig. 1f. To avoid interference of Si peak at 70°, the test range is close to 65°. Only two peaks appeared in the figure and can be indexed to CdS crystals (JCPDS file no. 41-1049), the absence of other peaks can prove that the product is a high-quality CdS crystal. Compared with the other peak of CdS, the sharp diffraction peak (002) at 26.253° indicates that the CdS nanorod array grows highly oriented along the CdS crystal *c*-axis. At the same time, we also studied the morphology characteristics of the deposited products without using the magnetron sputtering heating system, and the results further proved that the CdS nanorods we prepared are high-quality crystals (see Fig. S2 in ESI†). The energy band diagrams of the photodetector are shown in Fig. 1g. The valence and conduction bands of CdS and Si correspond to vacuum energy levels of −6.82 and −5.17 eV, −4.4 and −4.05 eV, respectively.^19,24^

### 3.2. Self-powered broadband response performances of p-Si/n-CdS nanorods array PDs

**Fig. 2:**
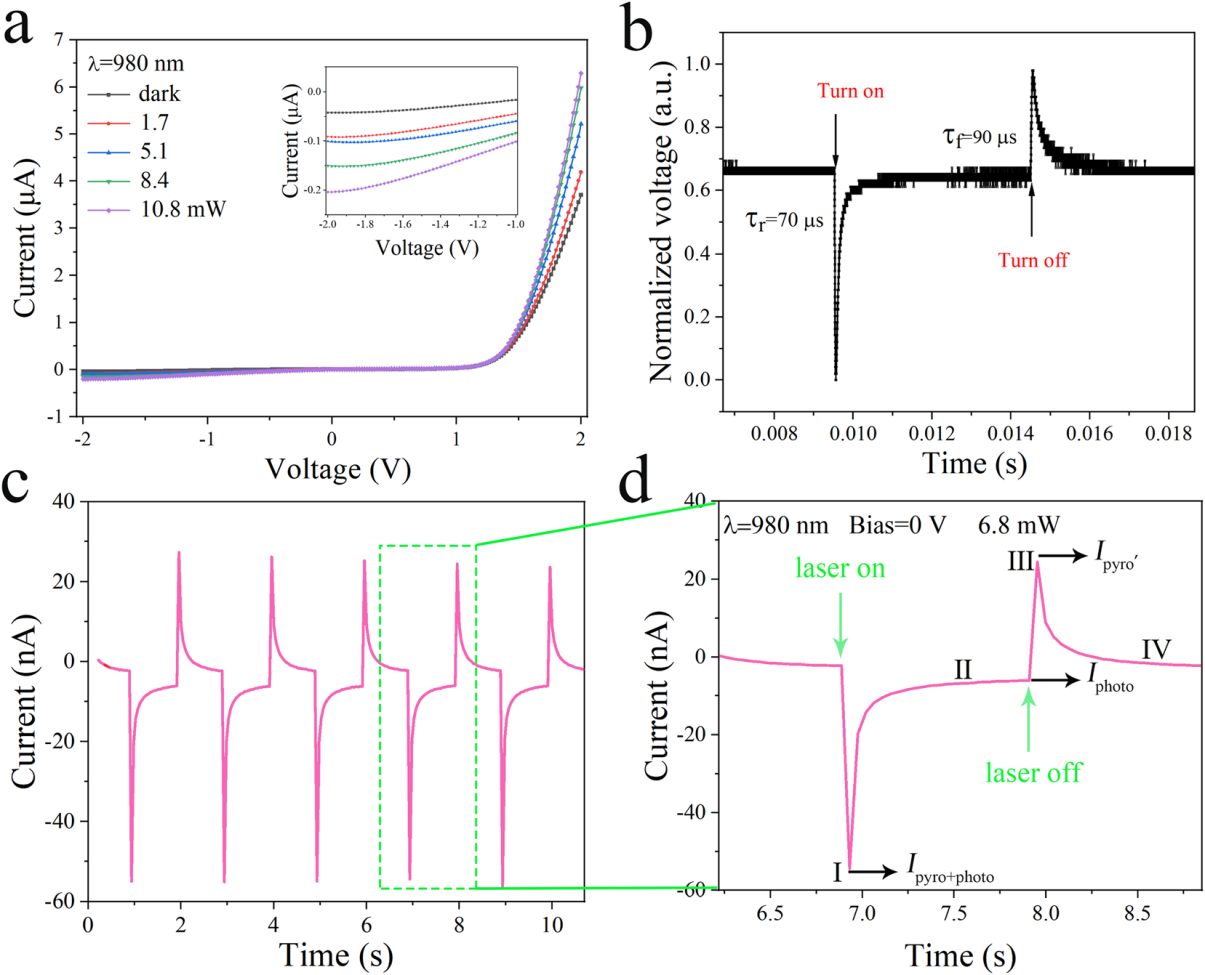
Photoelectric properties, photocurrent response, and working mechanism of the self-powered p-Si/n-CdS heterostructure PDs. (a) Steady-state *I*–*V* characteristics of PN junction PDs under 980 nm laser illumination at different powers from 0 to 10.8 mW when 2 V bias is applied. (b) The photocurrent response of a photodetector in a typical cycle of 980 nm pulsed light (100 Hz) at 0 bias voltage. (c) *I*–*t* characteristics of self-powered PDs under 980 nm laser illumination with the power of 6.8 mW at 0 V bias. (d) An enlarged view taken from the marked areas in (c), shows four-part photoresponse behavior upon exposure to a pulse of 980 nm light.

As shown in Fig. 2a, the photoelectric properties of the devices were investigated by plotting the current–voltage (*I*–*V*) relationship of the p-Si/n-CdS nanorods array PDs at steady-state, and the devices exhibit a significant rectification behavior in the absence of light due to the formation of a PN junction between CdS and Si. Under 980 nm laser irradiation, p-Si/n-CdS PDs exhibited obvious photoresponse behavior under 2 V forward bias, when the optical power increased from 0 to 10.8 mW, and the output current increased from 3.7 μA to 6.3 μA. When p-Si/n-CdS PDs are irradiated with 1310 nm laser light, due to the limited band gap of the material itself, photogenerated carriers will not be generated even under high light intensity irradiation, but pyroelectric charges are still generated by the pyroelectric effect, creating a pyroelectric field, and we can detect the current in an external circuit. During the measurement process, a laser light source with a fixed wavelength is used to generate high-speed pulsed laser light (980 nm, 100 Hz), the semiconductor analyzer measures the real-time changes, and the measured optical switching behavior is amplified to a period by connecting to an oscilloscope and the results depicted in Fig. 2b. From the details of the rise and decay processes within a single cycle, the rise and fall times are 70 μs and 90 μs, respectively. The pyro-phototronic effect has been proposed over the years to regulate the photoelectric process in photodetectors by coupling the pyroelectric effect and photoexcitation in pyroelectric semiconductors. As a typical II–VI semiconductor, CdS not only possesses excellent optoelectronic properties but also exhibits a pyroelectric effect due to its non-centrosymmetric crystal structure. The instantaneous increase in temperature of the material under illumination produces opposite pyroelectric charges at the −*c* and +*c* ends of the CdS nanorods, resulting in a pyroelectric field (*E*_pyro_) along the *c*-axis inside the CdS, which is equivalent to the electric field applied to a self-powered photodetector and in which we can detect the pyroelectric current in an external circuit.

Given this situation, we collected the photoresponse of the device through a semiconductor analyzer as shown in Fig. 2c, which exhibits a standard periodic variation. According to the principle of its photoresponse, a cycle can be divided into four parts and the amplified photoresponse of one cycle is plotted separately in Fig. 2d. In order to better understand the working mechanism of the photodetector, we draw a schematic diagram of the pyroelectric effect and the photoexcitation process (see Fig. S1 in ESI†). For the first part, due to the rise of the instantaneous temperature of CdS nanorods caused by laser illumination (d*T*/d*t* > 0), the pyro-phototronic effect will cause a sharp increase in current. It is obvious from the figure that the peak current is *I*_pyro+photo_, which indicates that the peak current is the result of both the pyroelectric and photovoltaic effects. For the second part, since there is no temperature change (d*T*/d*t* = 0), the pyro-phototronic effect is also absent, and the output current is greatly reduced back to a stable value, called the stable value of *I*_photo_, which means that the output current at this point only comes from the photovoltaic effect. For the third part, when the laser light source is turned off, the instantaneous temperature will drop (d*T*/d*t* < 0), and the output current will have a reverse peak *I*_pyro′_, where the reverse pyroelectric current *I*_pyro′_ and *I*_pyro_ are in opposite directions. For the fourth part, the pyroelectric current disappears when the CdS nanorods return to their initial temperature (d*T*/d*t* = 0) without laser irradiation.^15,18,34^ Under a certain frequency of the pulsed laser, the performance of *I*–*T* changes periodically.

**Fig. 3:**
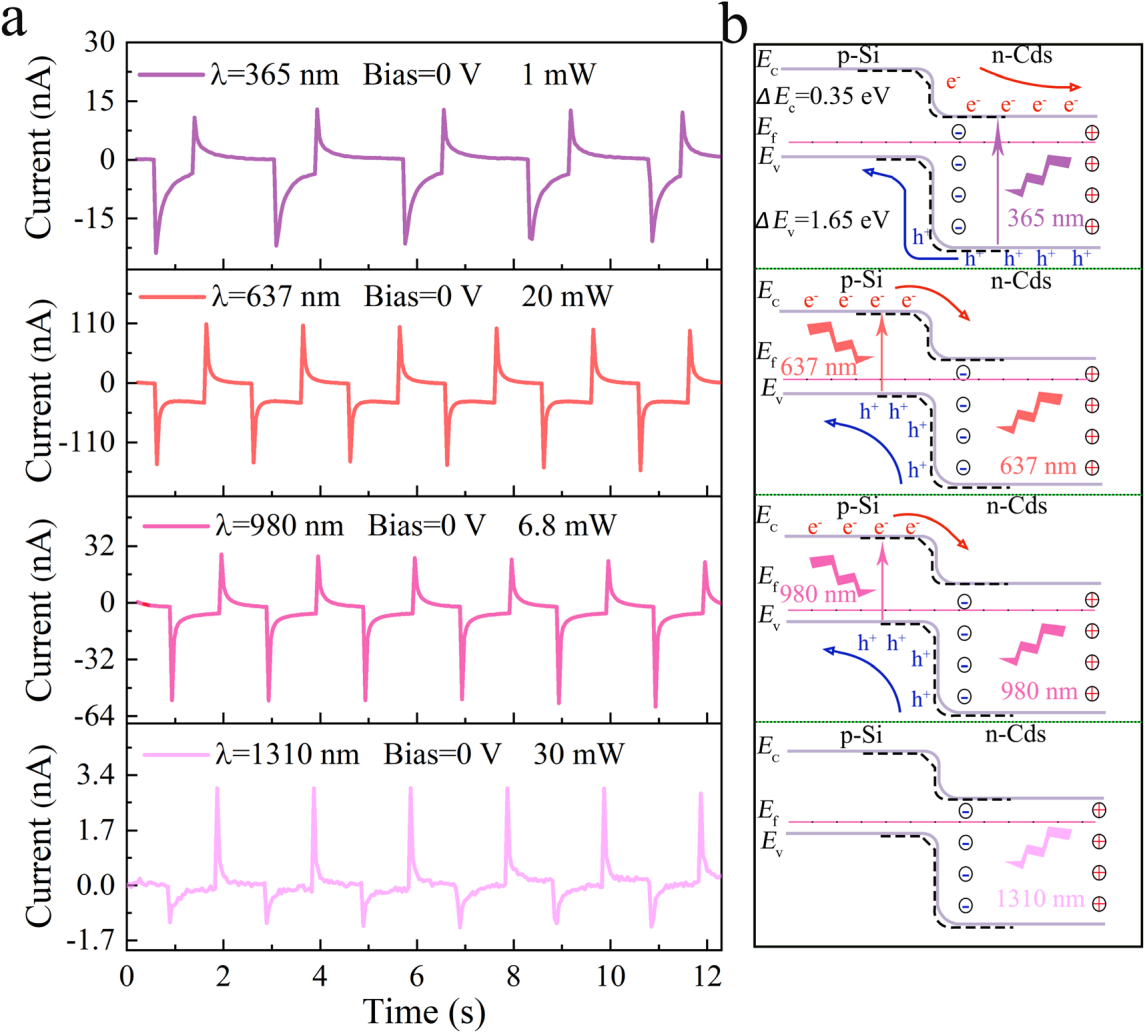
Broadband response of the self-powered p-Si/n-CdS heterostructure PDs. (a) *I*–*t* characteristics of self-powered PDs under 365, 637, 980, and 1310 nm laser illuminations at 0 V bias voltage. (b) The band diagram of the p-Si/n-CdS heterojunction interface under 365, 637, 980, and 1310 nm laser illuminations (black dotted lines indicate no illumination).

As far as the broadband photoresponse is concerned, the four-part response characteristics of the detector were investigated at several wavelength bands. Fig. 3a shows the results for devices irradiated with 365, 637, 980, and 1310 nm lasers, which exhibit detection capabilities from the UV to the IR. According to the four-part photoresponse behaviors shown in this figure, lasers with different wavelengths has different influences on self-powered PDs. The working mechanism of different wavelength irradiation is shown in Fig. 3b. When the device is illuminated, the inner CdS nanorods will heat up immediately to generate a temperature difference, which in turn induces the regulation of carriers by the built-in field at the PN junction interface. According to Anderson's model, the energy bands of CdS and Si will bend upwards (the dotted line is the initial state).^35^ As shown in the first part of Fig. 3a and the first part of Fig. 3b, ultraviolet photons are absorbed by CdS nanorods when irradiated by a 365 nm laser, and the electrons and holes generated in the CdS nanorods interact with the top and bottom electrodes respectively to generate *I*_photo_. At the same time, the pyroelectric potential and the upward bending of the energy band caused by the temperature change promote the transport of carriers, thereby obtaining the maximum instantaneous output current. As shown in the second and third parts of Fig. 3a and b, when irradiated with 637 nm and 980 nm lasers, visible photons and near-infrared photons are absorbed by the Si substrate, thus obtaining a larger *I*_photo_. It can be seen from the fourth part of Fig. 3a and b that when irradiated with a 1310 nm laser, both Si and CdS cannot generate photogenerated carriers because the band gap width of Si and CdS is exceeded. However, the pyroelectric effect, which is not limited by the absorption range, still leads to the generation of pyro-charges, forming *E*_pyro_, which achieves an instantaneous sharp change in the output current.

**Fig. 4:**
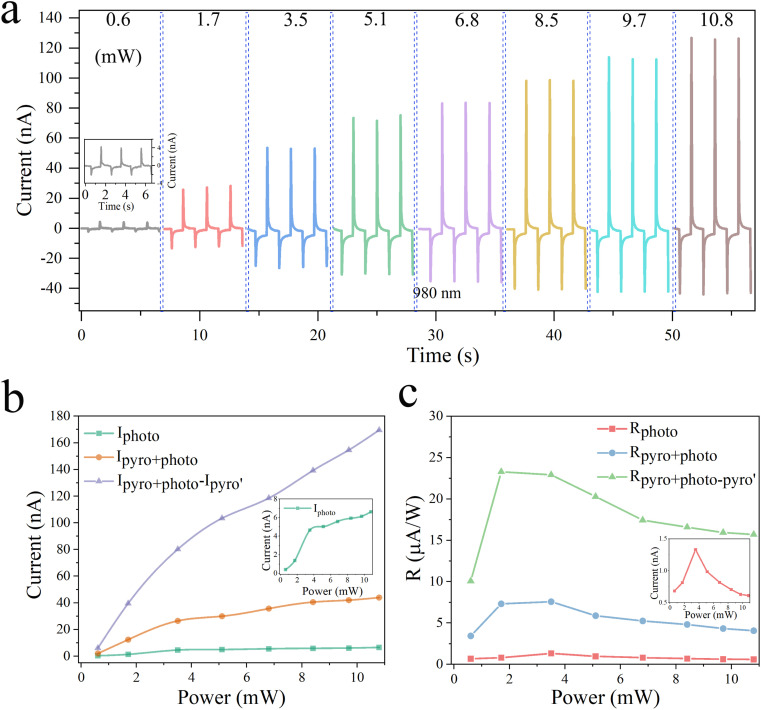
Pyro-phototronic effect on the self-powered p-Si/n-CdS heterojunction PDs under 980 nm laser illumination. (a) *I*–*t* characteristics of the self-powered PDs with different power from 0.6 to 10.8 mW. Inset: Enlarged *I*–*t* characteristics with a 0.6 mW power. (b) *I*_pyro+photo_, *I*_photo_, and *I*_pyro+photo_ − *I*_pyro′_ of the self-powered PDs as a function of the power. Inset: *I*_photo_ of the self-powered PDs as a function of the power. (c) Corresponding *R*_pyro+photo_, *R*_photo_, and *R*_pyro+photo−pyro′_ of the self-powered PDs as a function of the power. Inset: Corresponding *R*_photo_ of the self-powered PDs as a function of the power.

To study the synergistic working mechanism of the pyro-phototronic effect and photoelectric effect of self-powered PDs in detail, the four-part dynamic behavior and self-powered response were tested under laser irradiation at 980 nm with different optical powers, different bias voltage, and different frequencies. From Fig. 4a, it can be concluded that in the case of power measurement ranging from 0.6 to 10.8 mW and zero bias voltage, the measurement results all show an obvious four-part behavior, with a typical pyro-phototronic effect. At the same time, we plot the different current relationships corresponding to the values of the measured optical power in Fig. 4a, where *I*_photo_ means that the current is only generated by the photovoltaic effect; *I*_pyro+photo_ means that the current is a superposition of the pyroelectric current caused by the temperature rise and photocurrent generated by the photovoltaic effect; *I*_pyro′_ is the pyroelectric current caused by the temperature fall, and *I*_pyro+photo_–*I*_pyro′_ is the relative peak-to-peak current. It can be clearly seen from Fig. 4b that *I*_photo_, *I*_pyro+photo_, and peak current (*I*_pyro+photo_ − *I*_pyro′_) all increase with increasing illumination power. Through calculation and analysis, the maximum enhancement factors of (*I*_pyro+photo_ − *I*_pyro′_)/*I*_photo_ and *I*_pyro+photo_/*I*_photo_ are 28.2 and 8.9, under the illumination of 1.7 mW, respectively. To better explain the enhanced performance of self-powered PDs by the synergistic effect of the pyro-phototronic effect, a summary of the corresponding photoresponsivity *R* at different powers is presented in Fig. 4d. *R* is defined as 
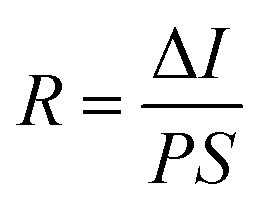
, where *P* is the intensity of incident light, *S* is the effective illumination area, Δ*I* = *I*_light_ − *I*_dark_, *I*_light_ is the photoresponse current, and *I*_dark_ is the dark current. D* is defined as 
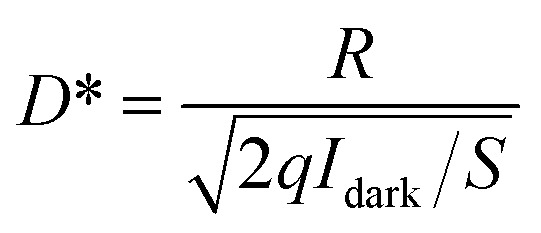
, describing the smallest signal that can be detected, where *q* is the electron charge.^36,37^ By analyzing the data, it is found that the photoresponsivity *R* corresponding to all powers is greatly enhanced by the pyro-phototronic effect. Under the laser irradiation of 980 nm, for the *I*_photo_ and *I*_pyro+photo_, the maximum photoresponsivity *R* is 1.33 and 7.58 μA W^−1^ when the optical power is 3.5 mW. For peak current (*I*_pyro+ photo_ − *I*_pyro′_), when the optical power is 1.7 mW, the maximum photoresponsivity *R* is 23.3 μA W^−1^.

**Fig. 5:**
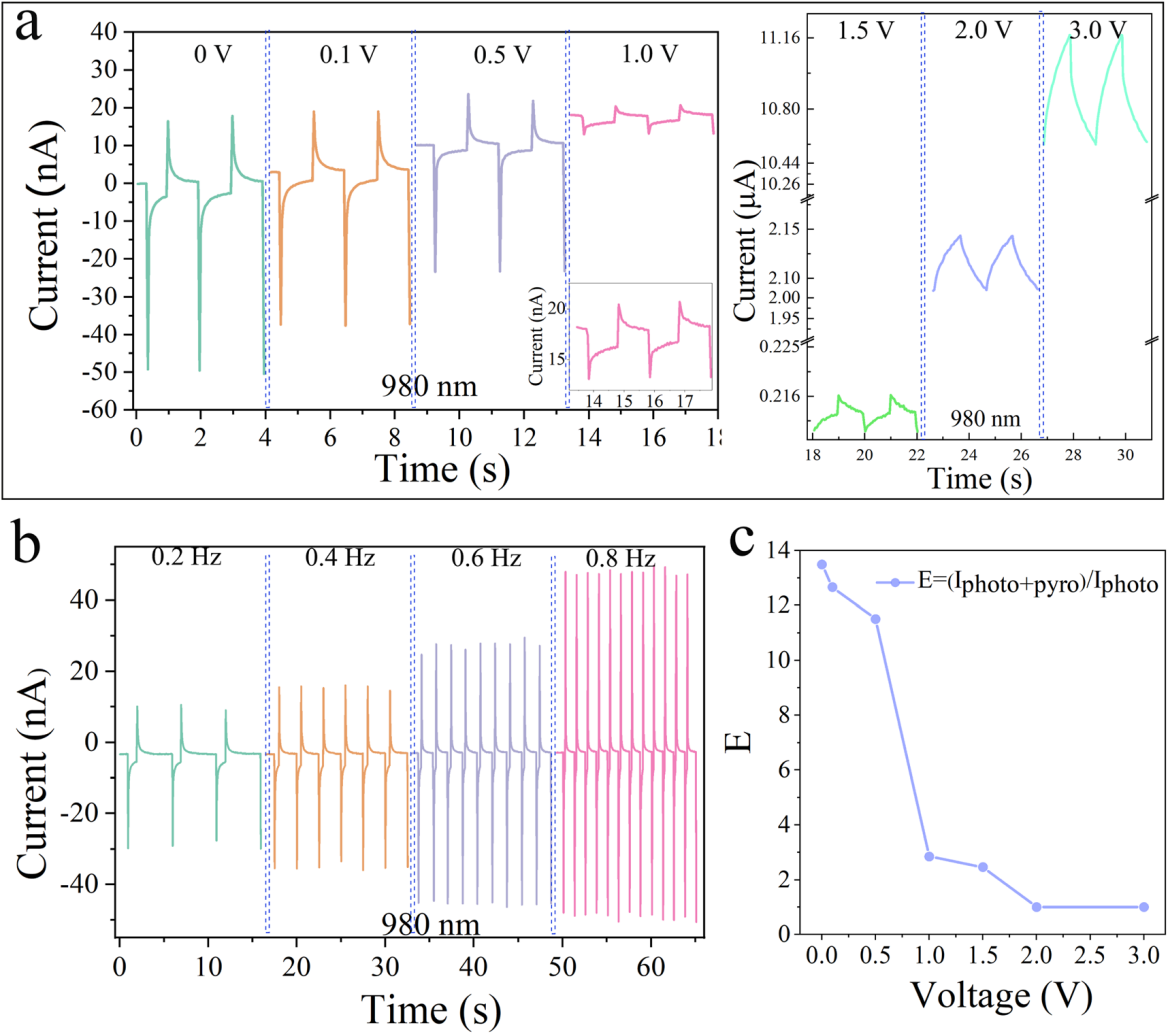
Photoresponse was measured under different bias voltages and frequencies under 980 nm laser illumination. (a) *I*–*t* characteristics of the self-powered p-Si/n-CdS heterojunction PDs under 980 nm laser illumination at different bias voltages from 0 to 3 V (power ≈ 10.8 mW). Inset: *I*–*t* characteristics of the self-powered p-Si/n-CdS heterojunction PDs under 980 nm laser illumination at 1 V bias voltages. (b) *I*–*t* characteristics of the self-powered p-Si/n-CdS heterojunction PDs under 980 nm laser illumination at different frequencies from 0.2 to 0.8 Hz (power ≈ 10.8 mW). (c) Corresponding enhancement factor *E* (= *I*_pyro+photo_/*I*_photo_) of the self-powered PDs as a function of bias voltage.

To further illustrate the photoresponse mechanism of the as-prepared photodetector, we investigated the effect of bias voltage on the photoresponse. The photodetector was illuminated with a laser with a power of 10.8 mW and a wavelength of 980 nm within a bias voltage of 0–3 V. It can be seen from Fig. 5a that the output current exhibits an obvious four-part dynamic behavior at a small bias voltage (less than 1 V), confirming the existence of pyro-phototronic effect CdS nanorods. However, as the bias voltage continues to increase, both photocurrent and dark current increase, and when the bias voltage is larger than 1 V, *I*_pyro′_ and *I*_pyro+photo_ gradually decrease until they disappear. The decreasing trend of the pyro-phototronic effect with increasing bias voltage is related to the emergence of joule heating, which is more deeply affected under the application of large bias voltage.^38,39^ As shown in Fig. 5c, an increase in bias voltage is accompanied by a decrease in *E* (= *I*_pyro+photo_/*I*_photo_), with *E* reaching a maximum of 13.48 at 0 V bias voltage and a minimum value at 3 V bias voltage, which proves that joule heating caused by the bias voltage weakens the pyro-phototronic effect. Fig. 5b shows the effects of different frequencies of laser light on the *I*–*t* characteristics of self-powered PDs under 980 nm laser irradiation with an optical power of 10.8 mW. It is obvious that *I*_photo_ is a frequency-independent characteristic that does not change. *I*_pyro_ shows a slight increase with a slight increase in frequency. When the laser is at a higher frequency, the time for illuminating the PDs device is shorter, and the corresponding temperature change rate (d*T*/d*t*) becomes larger.^33,39,40^ Therefore, within a certain range, *I*_pyro_ will increase with increasing frequency.

Although, the above results show that the pyroelectric effect can significantly improve the photodetection performance. In fact, not all semiconductors with wurtzite structure can exhibit obvious pyro-phototronics effect. In order to study the realization rule of pyro-phototronics effect, we have made a comparative experiment. In this experiment, a similar Si/CdS photodetector has been fabricated except that the CdS grown at room temperature. From the photoelectric test results in ESI,† the device exhibits excellent photodetection in bandgap range without apparent pyro-phototronics effect (see Fig. S3–S5 in ESI†). This is because when growing at room temperature, the atoms do not have enough energy to move freely, resulting in a decrease in the crystallinity of the film, which is not conducive to the generation of pyroelectric effects. Therefore, a reasonable material growth process is crucial to the realization of pyro-phototronics effect.

In general, CdS nanorods arrays have been prepared by magnetron sputtering to form self-powered PDs with Si substrate, which provides a good idea for the study of photo-induced pyro-phototronic effect in practical photodetection applications. From this work, it can be concluded that the p-Si/n-CdS nanorod heterojunction photodetector exhibits a broadband response from the UV (365 nm) to the IR (1310 nm), even beyond the bandgap limit of the material. This work not only analyzes the mechanism of different laser irradiation but also studies the influence of laser frequency and bias voltage on the pyro-phototronic effect. In addition, this work also analyzes the influence of the pyro-phototronic effect on the heterojunction, which provides an idea for designing a stable broadband response photodetector.^41–44^

## 4. Conclusion

In summary, we innovatively prepared wurtzite-structured CdS nanorod arrays by magnetron sputtering, combined the pyroelectric material CdS with Si, we finally designed a p-Si/n-CdS heterojunction broadband self-powered PDs enhanced by pyro-phototronic effect. In this work, our fabricated self-powered photodetector exhibits a broadband response (UV-IR) and ultrafast response speed (70 μs and 90 μs rise and fall times, respectively), with excellent photodetection performance. At the same time, the work also includes systematically studying the changes caused by the device being irradiated with several different wavelengths of laser light within the wavelength detection range, studying the influencing factors such as bias voltage and frequency, and analyzing its working principle. Furthermore, this work proposes and realizes synergy between optoelectronics and pyroelectric to enhance broadband responses, especially for wavelength bands beyond the material photoresponse bandgap. Therefore, this work also learns the role of the pyro-phototronic effect on heterojunction photodetectors and also provides an efficient method for designing low-cost, fast, and broadband-responsive self-powered PDs.

## Author contributions

Lu Li: investigation, design, software, experiment, data curation, writing. Ding-shan Zheng: discussion, wquipment. Yan Xiong: discussion, funding acquisition. Cheng Yu: discussion, characterization. Hong Yin: review, editing. Xiang-xiang Yu: investigation, design, writing, review, editing, funding acquisition.

## Conflicts of interest

The authors declare that they have no known competing financial interests or personal relationships that could have appeared to influence the work reported in this paper.

## Supplementary Material

RA-012-D2RA07314E-s001
