# Supplementary material for: Pyro-phototronic effect enhanced broadband photodetection based on CdS nanorod arrays by magnetron sputtering
Source: RSC Adv. 2022 Dec 9;12(54):35341–9. doi: 10.1039/d2ra07314e (PMC9733663; doi:10.1039/d2ra07314e)
Supplement: RA-012-D2RA07314E-s001 [file RA-012-D2RA07314E-s001.pdf]

Electronic Supplementary Material (ESI) for RSC Advances.
This journal is © The Royal Society of Chemistry 2022

Pyro-phototronic effect enhanced broadband photodetection based on CdS nanorod arrays by magnetron sputtering

Lu Li [a], Dingshan Zheng [a], Yan Xiong [a], Cheng Yu [b], Hong Yin [c, d*], Xiangxiang Yu [a*]

[a] *School of Physic and Optoelectronic Engineering, Yangtze University, Jingzhou 434023, P.R.China*

[b] *School of Geography Science and Geomatics Engineering, Su Zhou University of Science and Technology, No.99 Xuefu Road, SuZhou 215009, P.R.China.*

[c] *School of Chemistry and Chemical Engineering, Hunan Institute of Science and Technology, Yueyang 414006, PR China*

[d] *International Iberian Nanotechnology Laboratory (INL), Avenida Mestre Jose Veiga, 4715-330 Braga, Portugal*

*Corresponding Author: 2017507027@hust.edu.cn; yuxx518@126.com

For a better understanding of the working mechanism of the CdS/Si heterojunction photodetector, we have plotted a schematic diagram of the pyroelectric effect combined with the photoexcitation process in Fig. S1, which corresponds to the four stages marked in Fig. 2d.

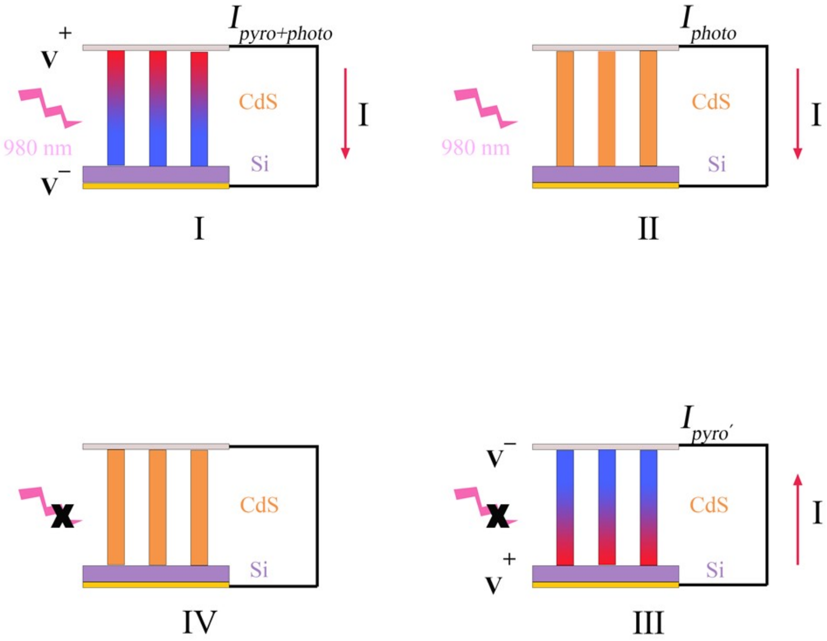

Fig. S1. Schematic illustration of the working mechanism of pyroelectric effect-combined photoexcitation processes, corresponding to the four stages labelled in figure 2d.

Notes: We add a set of experimental data, which differs from the experimental part in the manuscript is that the pressure of magnetron sputtering is 0.5 Pa, the sputtering power is 100 W, the sputtering time is 60 min, and the heating system for magnetron sputtering is not used.

Fig.S2a. and S2b show the top and cross-sectional field-emission scan electron microscopy (SEM) images of the CdS film. It can be seen that the surface of the CdS

film is flat and dense, showing small grains, and the cross-section shows an overall high degree of uniformity. However, by comparison with Fig.1b and 1c in the manuscript, it can be seen that the CdS film prepared without heating has a smaller grain size and does not show a cylindrical array of nanorods in the microstructure. Fig. S2c. shows the XRD pattern of the corresponding CdS film. By comparison with Fig. 1f in the manuscript it can be concluded that although the deposited products all show (002) oriented growth, the number of diffraction peaks is higher for the CdS film deposited without the use of the heating system, which is a side indication that the orientation of the CdS film is not exactly along one direction. A comparison of the heights of the peaks in the two plots shows that the height of the XRD diffraction peaks for the CdS nanorods obtained by heated deposition is much higher than when they were not heated.

I-V characteristics of the self-powered p-Si/n-CdS heterojunction under the dark condition and illumination with different intensities of 365 nm, 450 nm, 520 nm, 637 nm, and 980 nm lights are plotted in Fig. S3 and S4, respectively. Under dark conditions, the device exhibits significant rectification behaviors, indicating the formation of a PN junction photodiode. Interestingly we can observe that under different light intensities, the PDs works efficiently under negative applied voltage, while under forward bias the dark current and photocurrent are almost the same. This is consistent with the working principle of a typical photodiode. Under light from 365 nm to 980 nm, the PDs reveals clear photovoltaic behavior which originates from the built-in electric field at the CdS/Si PN junction. Additionally, it is observed that both the open circuit photovoltage and short circuit photocurrent increase with increasing

light intensity. We investigated the pulsed illumination of the device at different optical powers at 365 nm, 450 nm, 520 nm, 637 nm, and 980 nm, respectively, at zero bias voltage. As shown in Fig. S4 and S5, PDs exhibit stable and repeatable photocurrent pulses by repeatedly switching a light on and off.

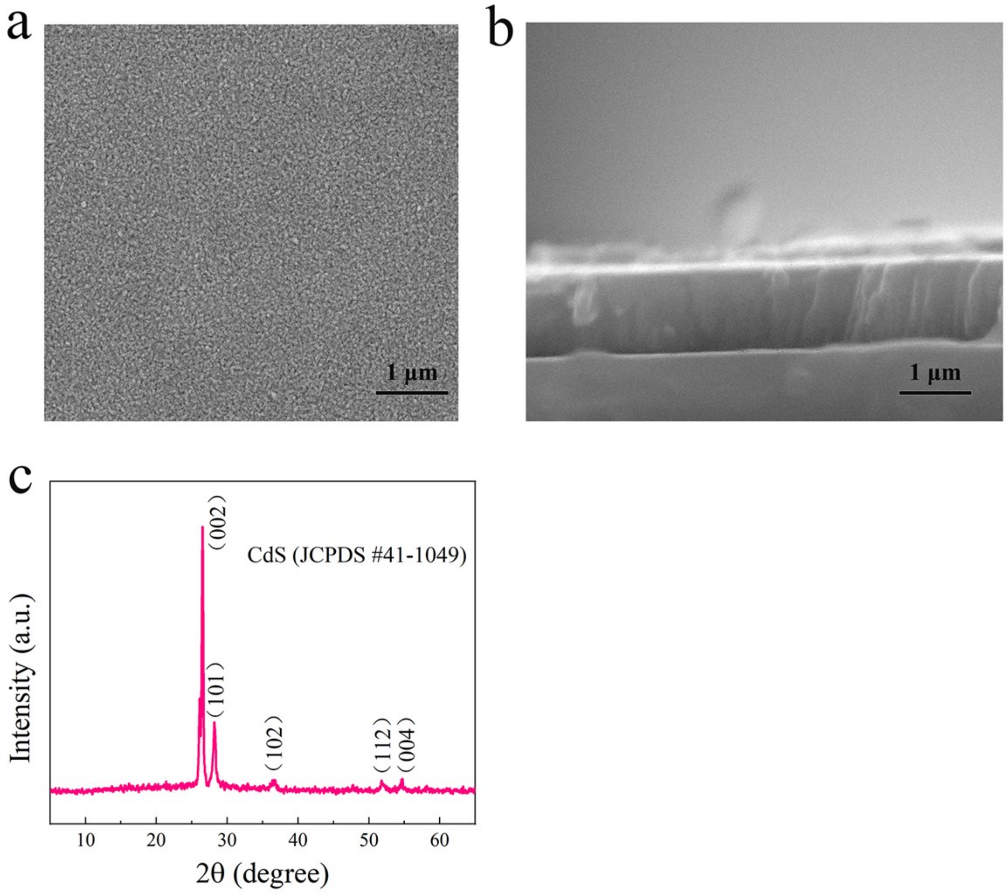

Fig. S2. Characterization of p-Si/n-CdS heterostructure PDs. (a-b) Top view and cross-sectional SEM image of the as-fabricated CdS film. (c) XRD pattern of CdS film.

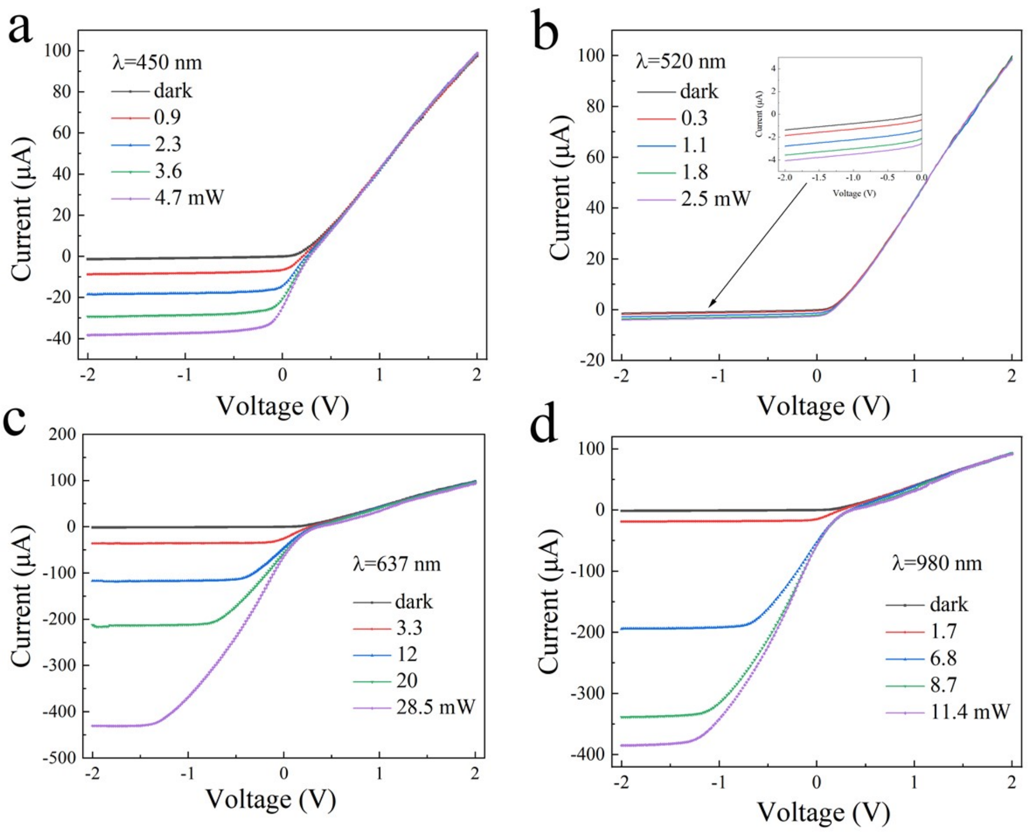

Fig. S3. (a-d) *I-V* characteristics of heterojunction PDs under 450 nm, 520 nm, 637 nm, and 980 nm light at different optical powers.

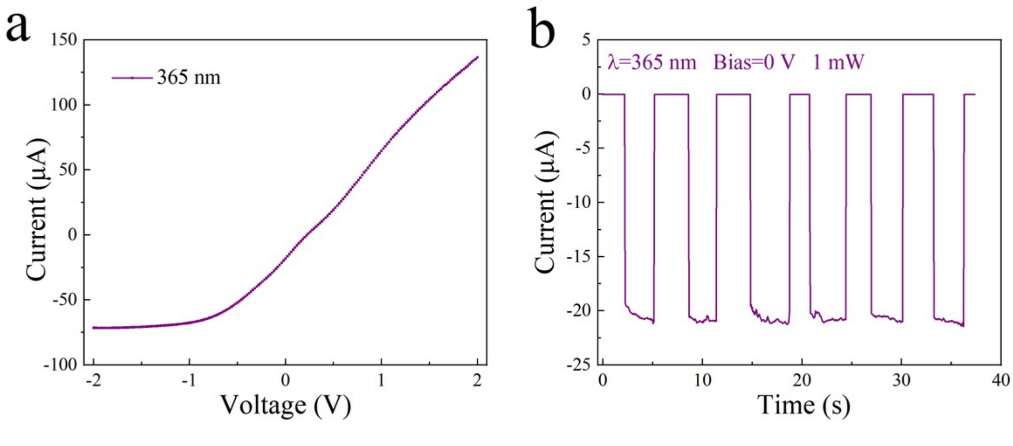

Fig. S4. (a-b) *I-V* characteristics and transient photocurrents of photodetectors under illumination at 365 nm.

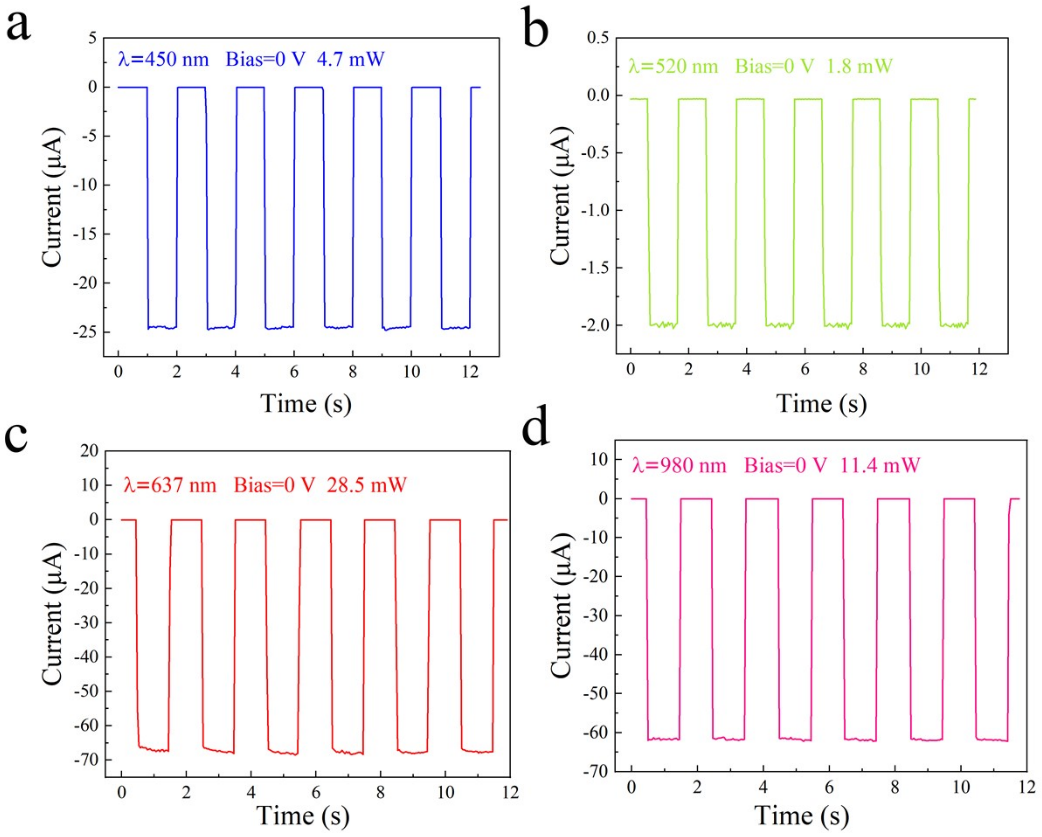

Fig. S5. Transient photocurrents under pulsed illumination of different intensities of 450 nm, 520 nm, 637 nm and 980 nm light.
